# Complete Genome Sequence of a New Zealand Isolate of the Bovine Pathogen Streptococcus uberis

**DOI:** 10.1128/genomeA.00119-18

**Published:** 2018-03-01

**Authors:** George Taiaroa, Nichaela Harbison-Price, Scott A. Ferguson, Glen P. Carter, Deborah A. Williamson, Richard C. Macknight, Gregory M. Cook, Adam Heikal

**Affiliations:** a Department of Microbiology and Immunology, University of Otago, Dunedin, New Zealand; b Department of Biochemistry, University of Otago, Dunedin, New Zealand; c Doherty Applied Microbial Genomics, Department of Microbiology and Immunology, The University of Melbourne at The Doherty Institute for Infection and Immunity, Melbourne, Australia; d Microbiological Diagnostic Unit Public Health Laboratory, Department of Microbiology and Immunology, The University of Melbourne at The Doherty Institute for Infection and Immunity, Melbourne, Australia; e Centre for Integrative Microbial Evolution (CIME), Faculty of Mathematics and Natural Sciences, University of Oslo, Oslo, Norway; f Laboratory for Microbial Dynamics (LaMDa), Section for Pharmaceutical Biosciences, School of Pharmacy, University of Oslo, Oslo, Norway

## Abstract

Streptococcus uberis forms part of the native microbiota of cattle and is able to opportunistically infect the mammary gland; as such, it is a leading cause of bovine mastitis globally. Here, we report the complete genome sequence of S. uberis NZ01, isolated in New Zealand from a cow with a clinical case of bovine mastitis.

## GENOME ANNOUNCEMENT

Streptococcus uberis is a Gram-positive bacterium with a global distribution, being commonly found as part of the native microflora in cattle (1, 2). Opportunistic infection of the bovine mammary gland by S. uberis can lead to the bacterium acting as a major pathogen, causing the inflammatory disease mastitis (3). Economic losses associated with bovine mastitis have been estimated at $35 billion per year globally, with S. uberis being the leading cause of bovine mastitis in New Zealand (4) and the United Kingdom (5) and a major cause in the Unites States (6), Canada (7), and Chile (8), among other countries.

As of this writing, there has been one complete genome of *S. uberis* assembled, which originated from the United Kingdom (2). Here, we report a second complete genome, that of New Zealand strain NZ01, which was isolated from a cow with a clinical case of bovine mastitis in Palmerston North, New Zealand. This complete genome will support efforts to manage bovine mastitis and further our understanding of the evolutionary responses of S. uberis to antimicrobial use.

DNA extraction and next-generation sequencing were performed at the Microbiological Diagnostic Unit Public Health Laboratory of the University of Melbourne. Genomic DNA was prepared from a culture grown from a single colony using a JANUS Chemagic workstation and Chemagic DNA/RNA kit (PerkinElmer, USA). DNA libraries were created using the Nextera XT DNA preparation kit (Illumina, USA). Next-generation sequencing was performed using the Illumina NextSeq platform.

The assembly involved a preliminary assembly in Geneious version 10.1.3 (9), followed by repeat spanning and gap closure with Sanger sequencing of PCR products. The complete genome was compared to that of S. uberis 0104J (GenBank accession number AM946015) by progressiveMauve alignment (10). Genomic features and coding DNA sequences (CDSs) were predicted with the NCBI Prokaryote Genome Annotation Pipeline (PGAP) (11).

The complete NZ01 genome comprises a single chromosome of 1,863,842 bp, with 1,808 CDSs and 1,886 predicted genes. Approximately 146 CDSs found in the NZ01 genome are novel, compared to those of strain 1040J from the United Kingdom. Conversely, approximately 163 CDSs found in the UK strain do not appear in NZ01, further illustrating the genomic flexibility of this species (12). Estimates of the S. uberis pangenome have been made on a geographically limited set of strains (13), with many of the above-described novel genes identified in NZ01 being found in this pangenome.

An identified prophage in NZ01 (1,520,084 to 1,560,140 bp) has been shown as active, with a subset of next-generation sequencing reads highlighting the presence of a circular phage particle. This could be of interest for the control of bovine mastitis, as the phage encodes multiple holin and lysin genes and may be able to actively lyse S. uberis (14).

As further efforts to control S. uberis are made, it will be increasingly useful to track the emergence of resistance to these measures, with complete genomes such as that presented here providing a resource for such investigations. A detailed phylogenetic study of this and other New Zealand S. uberis isolates will follow, with analysis of both horizontally acquired elements and antimicrobial resistance determinants.

### Accession number(s).

This complete genome sequence has been deposited at GenBank under the accession number CP022435.

## References

[1] Gilchrist TL, Smith DGE, Fitzpatrick JL, Zadoks RN, Fontaine MC 2013 Comparative molecular analysis of ovine and bovine *Streptococcus uberis* isolates. J Dairy Science 96:962–970. doi:10.3168/jds.2012-5705. 23200465

[2] Ward PN, Holden MT, Leigh JA, Lennard N, Bignell A, Barron A, Clark L, Quail MA, Woodward J, Barrell BG, Egan SA, Field TR, Maskell D, Kehoe M, Dowson CG, Chanter N, Whatmore AM, Bentley SD, Parkhill J 2009 Evidence for niche adaptation in the genome of the bovine pathogen Streptococcus uberis. BMC Genomics 10:54. doi:10.1186/1471-2164-10-54. 19175920 PMC2657157

[3] Bradley A 2002 Bovine mastitis: an evolving disease. Vet J 164:116–128. doi:10.1053/tvjl.2002.0724. 12359466

[4] McDougall S, Parkinson TJ, Leyland M, Anniss FM, Fenwick SG 2004 Duration of infection and strain variation in *Streptococcus uberis* isolated from cows’ milk. J Dairy Sci 87:2062–2072. doi:10.3168/jds.S0022-0302(04)70024-7. 15328218

[5] Bradley AJ, Leach KA, Breen JE, Green LE, Green MJ 2007 Survey of the incidence and aetiology of mastitis on dairy farms in England and Wales. Vet Rec 160:253–258. doi:10.1136/vr.160.8.253. 17322356

[6] Hogan JS, Smith KL 1997 Occurrence of clinical and sub-clinical environmental streptococcal mastitis. *In* Proceedings of the Symposium on Udder Health Management for Environmental Streptococci, p 59–75. Arlington, TX: National Mastitis Council, Inc.

[7] Vélez JR, Cameron M, Rodríguez-Lecompte JC, Xia F, Heider LC, Saab M, McClure JT, Sánchez J 22 5 2017 Whole-genome sequence analysis of antimicrobial resistance genes in *Streptococcus uberis* and *Streptococcus dysgalactiae* isolates from Canadian dairy herds. Front Vet Sci 4:63. doi:10.3389/fvets.2017.00063. 28589129 PMC5438997

[8] Reyes-Jara A, Cordero N, Aguirre J, Troncoso M, Figueroa G 2016 Antibacterial effect of copper on microorganisms isolated from bovine mastitis. Front Microbiol 7:626. doi:10.3389/fmicb.2016.00626. 27199953 PMC4848319

[9] Kearse M, Moir R, Wilson A, Stones-Havas S, Cheung M, Sturrock S, Buxton S, Cooper A, Markowitz S, Duran C, Thierer T, Ashton B, Meintjes P, Drummond A 2012 Geneious Basic: an integrated and extendable desktop software platform for the organization and analysis of sequence data. Bioinformatics 28:1647–1649. doi:10.1093/bioinformatics/bts199. 22543367 PMC3371832

[10] Darling AE, Mau B, Perna NT 2010 progressiveMauve: multiple genome alignment with gene gain, loss and rearrangement. PLoS One 5:e11147. doi:10.1371/journal.pone.0011147. 20593022 PMC2892488

[11] Tatusova T, DiCuccio M, Badretdin A, Chetvernin V, Nawrocki EP, Zaslavsky L, Lomsadze A, Pruitt KD, Borodovsky M, Ostell J 2016 NCBI Prokaryotic Genome Annotation Pipeline. Nucleic Acids Res 44:6614–6624. doi:10.1093/nar/gkw569. 27342282 PMC5001611

[12] Lang P, Lefébure T, Wang W, Zadoks RN, Schukken Y, Stanhope MJ 2009 Gene content differences across strains of *Streptococcus uberis* identified using oligonucleotide microarray comparative genomic hybridization. Infection, Genetics and Evolution 9:179–188. doi:10.1016/j.meegid.2008.10.015. 19056519

[13] Hossain M, Egan SA, Coffey T, Ward PN, Wilson R, Leigh JA, Emes RD 2015 Virulence related sequences; insights provided by comparative genomics of *Streptococcus uberis* of differing virulence. BMC Genomics 16:334. doi:10.1186/s12864-015-1512-6. 25898893 PMC4427978

[14] Celia LK, Nelson D, Kerr DE 2008 Characterization of a bacteriophage lysin (Ply700) from Streptococcus uberis. Vet Microbiol 130:107–117. doi:10.1016/j.vetmic.2007.12.004. 18242012

